# MicroRNA Variants and HLA-miRNA Interactions are Novel Rheumatoid Arthritis Susceptibility Factors

**DOI:** 10.3389/fgene.2021.747274

**Published:** 2021-10-29

**Authors:** Shicheng Guo, Yehua Jin, Jieru Zhou, Qi Zhu, Ting Jiang, Yanqin Bian, Runrun Zhang, Cen Chang, Lingxia Xu, Jie Shen, Xinchun Zheng, Yi Shen, Yingying Qin, Jihong Chen, Xiaorong Tang, Peng Cheng, Qin Ding, Yuanyuan Zhang, Jia Liu, Qingqing Cheng, Mengru Guo, Zhaoyi Liu, Weifang Qiu, Yi Qian, Yang Sun, Yu Shen, Hong Nie, Steven J. Schrodi, Dongyi He

**Affiliations:** ^1^ Department of Medical Genetics, School of Medicine and Public Health, University of Wisconsin-Madison, Madison, WI, United States; ^2^ Shanghai University of Traditional Chinese Medicine, Shanghai, China; ^3^ Department of Rheumatology,Guanghua Hospital Affiliated to Shanghai University of Traditional Chinese Medicine, Shanghai, China; ^4^ Department of Health Management, Shanghai East Hospital, Tongji University School of Medicine, Shanghai, China; ^5^ Institute of Arthritis Research in Integrative Medicine, Shanghai Academy of Traditional Chinese Medicine, Shanghai, China; ^6^ Shanghai Institute of Immunology, Shanghai Jiao Tong University School of Medicine, Shanghai, China

**Keywords:** rheumatoid arthritis, miRNA, SNP, susceptibility, predispose

## Abstract

Genome-wide association studies have identified >100 genetic risk factors for rheumatoid arthritis. However, the reported genetic variants could only explain less than 40% heritability of rheumatoid arthritis. The majority of the heritability is still missing and needs to be identified with more studies with different approaches and populations. In order to identify novel function SNPs to explain missing heritability and reveal novel mechanism pathogenesis of rheumatoid arthritis, 4 HLA SNPs (*HLA-DRB1*, *HLA-DRB9*, *HLA-DQB1,* and *TNFAIP3*) and 225 common SNPs located in miRNA, which might influence the miRNA target binding or pre-miRNA stability, were genotyped in 1,607 rheumatoid arthritis and 1,580 matched normal individuals. We identified 2 novel SNPs as significantly associated with rheumatoid arthritis including rs1414273 (*miR-548ac*, OR = 0.84, *p* = 8.26 × 10^−4^) and rs2620381 (*miR-627,* OR = 0.77, *p* = 2.55 × 10^−3^). We also identified that rs5997893 (*miR-3928*) showed significant epistasis effect with rs4947332 (*HLA-DRB1*, OR = 4.23, *p* = 0.04) and rs2967897 (miR-5695) with rs7752903 (*TNFAIP3*, OR = 4.43, *p* = 0.03). In addition, we found that individuals who carried 8 risk alleles showed 15.38 (95%CI: 4.69–50.49, *p* < 1.0 × 10^−6^) times more risk of being affected by RA. Finally, we demonstrated that the targets of the significant miRNAs showed enrichment in immune related genes (*p* = 2.0 × 10^−5^) and FDA approved drug target genes (*p* = 0.014). Overall, 6 novel miRNA SNPs including rs1414273 (*miR-548ac*, *p* = 8.26 × 10^−4^), rs2620381 (*miR-627*, *p* = 2.55 × 10^−3^), rs4285314 (miR-3135b, *p* = 1.10 × 10^−13^), rs28477407 (miR-4308, *p* = 3.44 × 10^−5^), rs5997893 (*miR-3928*, *p* = 5.9 × 10^−3^) and rs45596840 (*miR-4482*, *p* = 6.6 × 10^−3^) were confirmed to be significantly associated with RA in a Chinese population. Our study suggests that miRNAs might be interesting targets to accelerate understanding of the pathogenesis and drug development for rheumatoid arthritis.

## Background

Rheumatoid arthritis (RA) is a chronic inflammatory disorder caused by the interaction between multiple factors including genetics, epigenetics, and the environment (van der Woude et al., 2010; Stahl et al., 2012; Cavalli and Heard, 2019). Twin studies estimate an RA heritability of ∼60% (Eyre et al., 2012; Westra et al., 2018). In the past 15 years, GWAS have identified several hundred RA-associated variants. However, the reported genetic variants only explain <40% of RA heritability (Stahl et al., 2010; Okada et al., 2012; Raychaudhuri et al., 2012; Westra et al., 2018). Furthermore, studies within non-European-derived samples are needed to aid understanding of RA molecular pathophysiology as different variants and, potentially, different disrupted pathways may be present in those sample sets. Additionally, studying SNPs in specific functional classes of genes or motifs may reveal specific pathogenic mechanisms not previously discovered through GWAS (Li et al., 2018). GWAS results indicate that 90% of disease-associated variants are located in non-coding regions, indicating that regulatory elements may play important roles in complex disease etiology, including RA (Maurano et al., 2012). Across regulatory elements, microRNAs are important targets to interrogate as miRNA SNPs, which may modify the expression of numerous genes. Moreover, miRNAs play pivotal roles in both innate and adaptive immunity (Lai et al., 2017; Zhu et al., 2020), sex-specific effects (Khalifa et al., 2016), and disease onset. (Chatzikyriakidou et al., 2012; Moran-Moguel et al., 2018; Karami et al., 2020). The interaction of the proteins involved in antigen presentation, such as HLA class I proteins have been extensively studied in which interactions between the alleles of the HLA haplotypes were found to affect the immune response levels and autoimmune disease susceptibility (Shiina et al., 2009). However, the interaction between HLA-genes with non-HLA regions, especially epigenetic factors like miRNA has not been widely investigated.

Recently, several preliminary RA-association studies focused on miRNAs have been conducted for Asian (Yang et al., 2011; Yang et al., 2012; Li et al., 2014; Fu et al., 2016; Yang et al., 2017), European (Hashemi et al., 2013), and African (Shaker et al., 2018) populations. However, these studies have very limited sample sizes and only included a subset of miRNAs without genotyping all miRNA common SNPs. Additionally, the previous RA GWAS in Han Chinese was conducted in 2014 (Jiang et al., 2014) using an array with poor coverage of miRNA SNPs. Therefore, we conducted an exhaustive study of functional miRNA SNPs in RA. miRbase annotated 1,920 primary miRNA and 2,883 mature miRNAs. From dbSNP153, there are >45,705 SNPs located in primary miRNAs and 17,570 in mature miRNA (Kozomara et al., 2019); however, only 733 of these SNPs segregate alleles at a moderate-high frequency (i.e. are common SNPs with MAF>1%) within primary miRNAs regions. Within specific populations, this number is expected to be substantially reduced to ∼200 SNPs, providing an opportunity to use a multiplex genotyping assay in our RA samples. Hence, we conducted a systemic association study between common East-Asian miRNA SNPs with RA in a large Han Chinese cohort to identify novel miRNA SNPs and miRNA epistatic interactions.

## Methods

### Precision Medicine Research Cohort in Shanghai Guanghua Hospital

Guanghua Hospital Precision Medicine Research Cohort (PMRC) is a hospital-based longitudinal cohort to investigate risk factors, genetic susceptibility, pharmacogenetics for rheumatology diseases such as RA, osteoarthritis, and ankylosing spondylitis. Healthy individuals are derived from those with an annual physical exam without rheumatological disease. Currently, PMRC has enrolled >30,000 disease patients and 10,000 healthy individuals as controls. We randomly selected 3,223 individuals including 1,625 seropositive (RF+ and anti-CCP+) RA and 1,598 controls from the PMRC. Written consent was collected prior to enrollment. Non-Han Chinese individuals were excluded from the study to avoid confounding by population stratification. All cases fulfilled the 2010 European League against Rheumatism–American College of Rheumatology criteria or 1987 American College of Rheumatology revised criteria for RA. All healthy controls were required do not have personal or family history of ankylosing spondylitis, rheumatoid arthritis, osteoarthritis, type 1 and 2 diabetes, chronic infection, or common cancers. We did not find a significant case/control difference between gender, smoking, drinking, BMI (1,607 RA and 1,580 normal individuals) while the RA cohort shows a slightly higher age compared with the normal cohort (*p* = 0.035). We adjusted the above confounder in our association test between SNPs and RA traits.

The study was reviewed and approved by the Institutional Review Board of Guanghua Hospital (No: IRB12018-K-12) and all methods were performed in accordance with the relevant guidelines and regulations. The demographic and clinical characteristics of the whole sample are presented in Supplementary Table S1.

### DNA Extraction, SNP Selection, and Genotyping

Genomic DNA was extracted from peripheral blood using the CB-kit (CoWin Biosciences, CWBIO., China) following the manufacturer’s instructions. Allelic discrimination was automated using the manufacturer’s software, which has been widely described in previous studies (Jiang et al., 2012). Internal positive and negative control samples were used and a test of Hardy-Weinberg equilibrium was employed to assess the genotyping quality (SNPs exceeding HWE *p*-value cutoff were excluded from RA association testing). Cases and controls were required to have a family history of at least three generations of residency in Shanghai or neighboring regions. The SNaPshot Multiplex System (Applied Biosystem, USA) was used for the genotyping which has been widely used in our previous studies (Huang et al., 2014; Shen et al., 2016).

**FIGURE 1:**
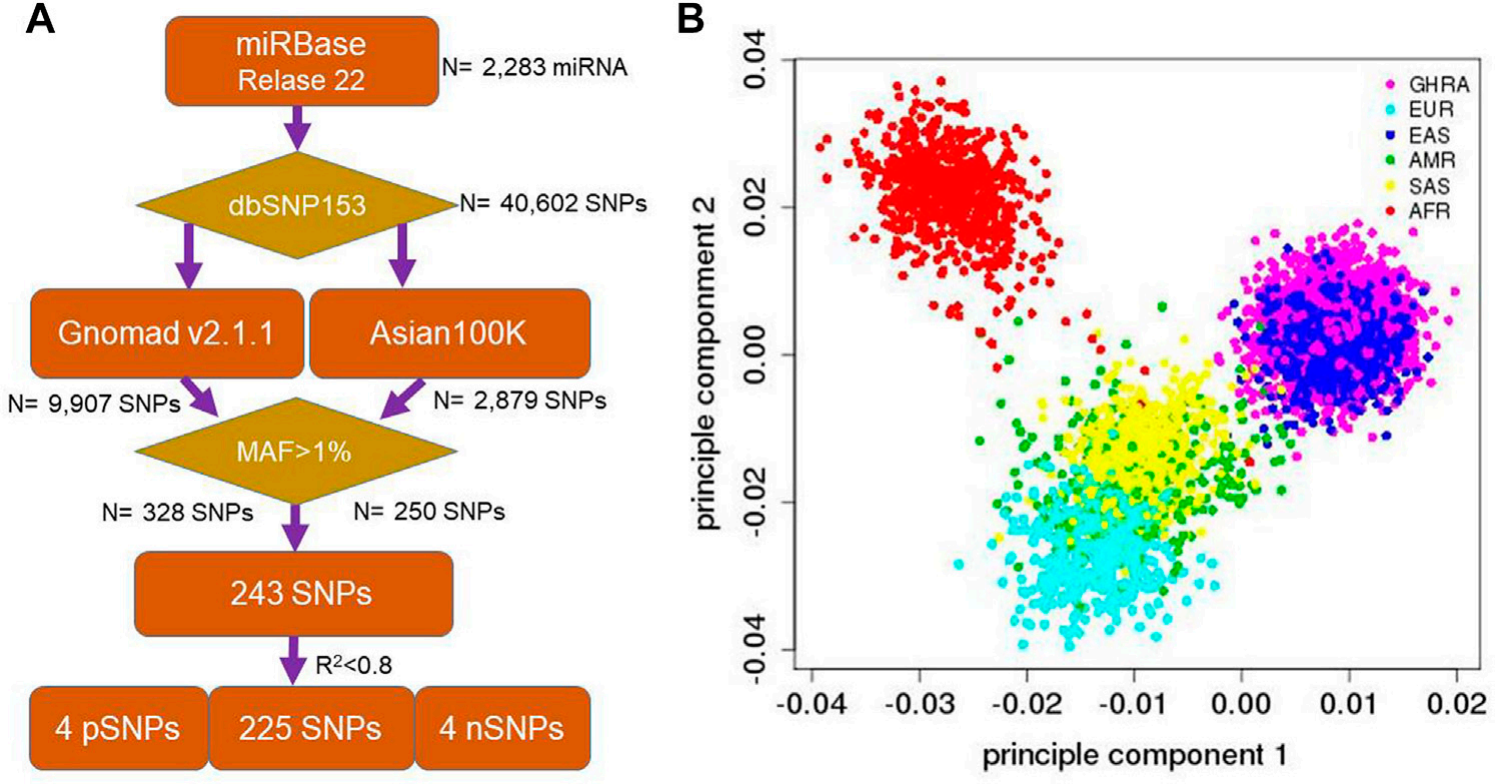
Flowchart for SNP and sample selection as well as population ancestry of GHRA cohort. **(A)** human microRNAs were downloaded from miRBase (Release 22.1). We mapped all the dbSNP153 SNPs into human microRNA genomic regions and 40,602 SNPs were received. We filter out non-East common variants with Gnomad and Asian 100K data with MAF>1%. 4 positive control (*HLA-DRB1*, *HLA*-*DRB9*, *HLA*-*DQB1,* and *TNFAIP3*) and 4 ancestry markers (South and North Han Chinese) were added for the quality control **(B)** PCA analysis to show the population ancestry of Guanghua Rheumatoid Arthritis cohort (GHRA) with 1,000 Genome dataset (included in dbSNP153). Symbols are as same as Super Population Code (SPC) from the 1,000 genome project. AFR, African; AMR, Ad Mixed American; EAS, East Asian; EUR, European; SAS, South Asian.

To select SNPs for subsequent association testing, human microRNAs were downloaded from miRBase methods (Release 22.1). Mapping of SNPs within dbSNP (dbSNP153, 08/08/2019) was performed for human microRNA genomic regions, resulting in 40,602 SNPs. To select common SNPs (MAF>0.01), we collected the allele frequency for all the SNPs from Gnomad (Lek et al., 2016), Asian100K (GenomeAsia100K Consortium, 2019), and the 1,000 Genomes dataset (Maurano et al., 2012) The minor allele frequency was required to be higher than 0.01 in all datasets (1000 Genomes Project Consortium et al., 2015). Only biallelic SNPs were included in the analysis, while tri-allelic SNPs were excluded from this study. Following filtering, 243 SNPs were obtained. In order to decrease the genotyping cost, one SNP from pairs of SNPs with high LD (R^2^ > 0.8) were removed and only one of them was included in the genotyping assay. Four SNPs within known GWAS associations with RA were included: rs9268839 (*HLA*-*DRB9*) (Okada et al., 2014; Laufer et al., 2019), rs9275376 (HLA-DQB1) (Regueiro and Gonzalez, 2020; Guo et al., 2019; Song et al., 2014; Castro et al., 2001; van Kerckhove et al., 1991), rs4947332 (tag-SNP for HLA-DRB1*04:07) and rs7752903 (*TNFAIP3*) (Okada et al., 2014; Laufer et al., 2019) In addition, a panel of 4 ancestry informative markers (negative control) were selected to estimate the potential effects of population stratification: rs174583 (11q12.2), rs11745587 (5q31.1), rs521188 (1p31.3) and rs7740161 (6q23.2). These SNPs were derived from a previous Han Chinese population study (Qin et al., 2014) and are informative in comparing the population structure between South and North Han Chinese since the Shanghai region is a mixed population from different regions in China (Figure 1A). Finally, a total of 233 SNPs were genotyped and 225 common miRNA SNPs were included in the novel RA-association SNP identification study.

### Quality Control and Power Analysis

For genotyping QC, ∼2.5% samples (40 RA and 32 normal) were randomly selected for repeat genotyping. The concordance between initial and replicate genotyping was 99.98%. SNP imputation was accomplished through the Michigan Imputation Server (https://imputationserver.sph.umich.edu/index.html) with East Asian samples from the Genome Asian Pilot (GAsP) since recent evidence shows that the GAsP reference panel showed higher imputation accuracy (93–95%) compared with 1,000 Genome Asian panel (<90%) (Belsare et al., 2019). The missing ratio between case and control differential test and HWE in control samples were evaluated. SNPs were removed when *p* < 0.01. GRCH37/hg19 was used to determine genomic positions and R^2^ > 0.6 as the cut-off to selected imputed SNPs of high quality and located in miRNA regions.

In the power analysis, given the prevalence of RA (*p* = 0.01), case (*n* = 1,607) and control (*n* = 1,580) size, risk allele frequency (f = 0.1) and genetic effect size (OR = 1.5), the power for multiplicative model, additive model, dominant models were 0.856, 0.823 and 0.742, respectively using a significance level of alpha = 5 × 10^−3^. In addition, with our design and the above effect size, we calculate that SNPs with an MAF of 0.025 can attain a statistical power of 80% with nominal *p* < 0.05. Therefore, our study could provide reliable genetic association results for the majority of the alleles we have included given that the MAF of 201 (86.3%) SNPs are >10% and MAF of 96% included SNPs exceed 2.5%.

### Association Analysis, Power Calculations, Epistasis Analysis, and Cumulative Risk Analysis

The association for each SNP was calculated and OR estimated (95% CI) using Chi-square test, Fisher’s Exact test (N < 5), Cochran-Armitage Trend test, and Bayesian logistic regression (BLR) (Banerjee et al., 2018) adjusted for gender (binary), age (continuous), BMI (continuous), smoking (binary) and drinking history (binary), using the bayesm (version: 3.1.4) package (Banerjee et al., 2018). To explore the power of the study, a Monte Carlo simulation was performed under different models including dominant, recessive, and additive models, additive models, using a Chi-square test or Bayesian logistic regression model. We performed fixed-effects and random-effects meta-analysis with plink between our dataset and Okada’s dataset (Okada et al., 2014) to identify novel RA-associated miRNA-SNPs by large sample size and statistical power. SNP-SNP interactions were analyzed by using traditional point-wise interaction analysis based on SNPassoc (Landa et al., 2009), logistic test (Boulesteix et al., 2007), “fast-epistasis” in Plink (Purcell et al., 2007). We applied Y ∼ b0 + b1*A+ b2*B+ b3*AB+ e to detect epistasis effect between SNP A and SNP B when b3 not equal 0 (null hypothesis: b3 equal 0). A *p* < 0.001 was considered significant. A cumulative RA risk prediction model was fitted with the 6 most significant SNPs (rs1414273, rs4947332, rs9268839, rs9275376, rs7752903, and rs2620381) by both a Chi-square and logistic regression test to adjust by the aforementioned covariates and in which individuals carrying 1 risk allele were used as the reference. Multiple test correction was conducted through a False Discovery Rate (FDR) implemented in R with the function p. adjust) (Ghosh, 2019). SNP derived miRNA target gain or loss were imputed by miRNA-SNP database (Gong et al., 2015) with the default setting. All the statistical analyses were performed with R (version: 3.6.1).

### Bioinformatics Analysis of miRNA Regulatory Networks and Enrichment Analysis

To understand the regulatory networks driven by miRNAs and the overlap with RA GWAS genes and immune system genes, a miRNA regulatory network analysis was performed with miRDB (Chen and Wang, 2020). Genes predicted to be regulated by significant miRNAs were calculated among 123 previously-identified RA GWAS genes, 4,723 immune system genes collected by InnateDB database (Breuer et al., 2013), and 373 FDA-targeted pharmacogenes. A target sore threshold was set to 95 in miRDB to identify reliable gene targets for the miRNAs. Cytoscape was employed to construct and visualize the miRNA-mRNA network. We calculated a hypergeometric test of the enrichment in miRNA targets within RA GWAS significant genes, immune-related genes, and FDA approved drug targets. A random sampling technique was applied to assess the null distribution of *p*-values (*n* = 50,000 iterations). 37,875 total genes in the genome (GENCODE v32) were assumed for the calculations. Enrichment was measured with fold-change (FC) and *p*-values were calculated by summing the frequency of more extreme values in the null distribution of *p*-values.

## Results

### SNP Selection, Genotyping, and Population Structure

Overall, the genotyping generated high quality data with 94% of the SNPs showing high genotyping quality. Seven of the SNPs were removed from being promoted to the RA association evaluation due to them having a genotyping ratio of less than 99.0%. The missing ratio between case and control was calculated. Hardy-Weinberg equilibrium in control samples was evaluated and another 3 SNPs were removed when *p* < 0.01. Finally, 223 SNPs in 1,607 rheumatoid arthritis 1,580 normal individuals were used in the association analysis (genotyping rate = 98.88%). PCA analysis based on these SNPs showed our samples clustered with the East Asian population (Figure 1B). Furthermore, we did not identify significant age, BMI, drinking, and smoking history differences between cases and controls (*p* > 0.01, Supplementary Table S1). However, as a conservative measure, association testing of all the variants included age, gender, BMI, drinking, and smoking as covariates in the Bayesian logistic regression (BLR)-based tests.

### Novel RA-Associated SNPs Located in *miRNA-548* and *miRNA-627*

**FIGURE 2:**
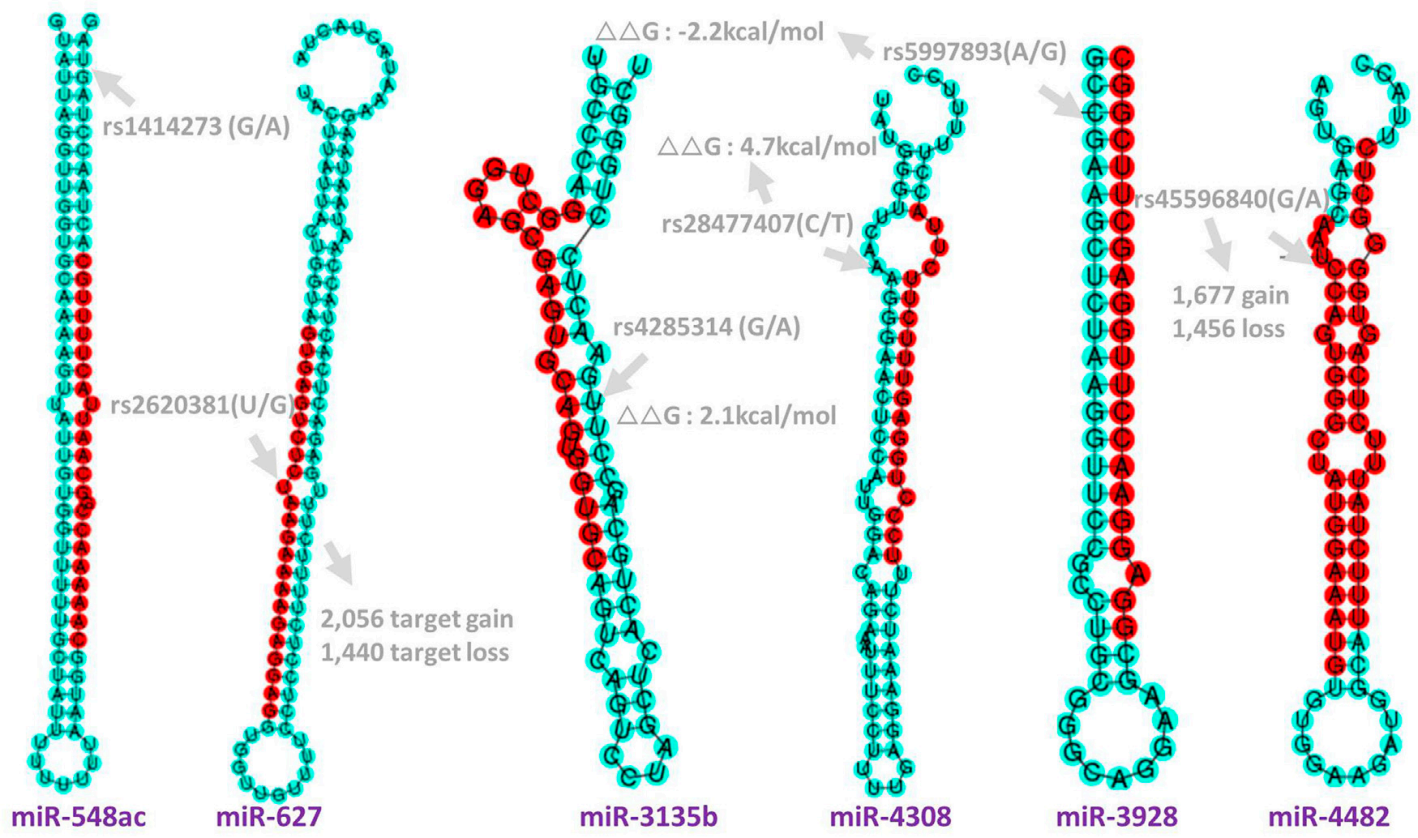
Genomic position and functional annotation to the significant SNPs in miRNAs. rs1414273 (C/T) in *miR-548ac*; rs2620381 (A/C) in miR-627*-5p* which cause 2,056 target gain and 1,440 target loss. rs4285314 (G/A) in miR-3135b; rs28477407(C/T) in *miR-4308*; rs5997893 (A/G) in *miR-3928-5p* and rs45596840 (G/A) in *miR-4482-5p* which will cause 1,677 target gain and 1,456 target loss. Target gain or loss were imputed by miRNA-SNP database (Gong et al., 2015) (see method).

Applying the Bayesian logistic regression model adjusted for covariates, we identified 6 significant SNPs located in *HLA-DRB9* (rs9268839, *p* = 3.95 × 10^−27^), *HLA-DRB1* (rs4947332, *p* = 2.78 × 10^−4^), *HLA-DQB1* (rs9275376, *p* = 2.65 × 10^−20^), *TNFAIP3* (rs7752903, *p* = 2.33 × 10^−4^), *miR-548ac* (rs1414273, *p* = 8.26 × 10^−4^) and *miR-627* (rs2620381, *p* = 2.55 × 10^−3^) and 15 additional SNPs with *p* < 0.05 (Table 1). As expected, all the SNPs located in *HLA-DRB1*, *HLA-DRB9, HLA-DQB1,* and *TNFAIP3* were significantly associated with RA status, which is consistent with previous GWAS studies. We found all the population markers including rs174583 (FDR = 0.88), rs11745587 (FDR = 0.98), rs521188 (FDR = 0.99), and rs7740161 (FDR = 0.48) were not significant in the association test between RA and control. Additionally, two miRNA SNPs located in *miR-548ac* (FDR = 0.01) and *miR-627* (FDR = 0.045) were significantly associated with RA after FDR adjustment for multiple testing (*p* < 0.05, FDR). We also conducted mode of inheritance-based association analyses (Table 2) and Cochran-Armitage Trend test-based association analyses (Supplementary Table S2). We found a dominant model of two miRNA SNPs (rs1414273 and rs2620381) that showed a highly significant association between the alleles and RA status. To combine previous Han Chinese association data with this study, we also implemented a meta-analysis in which 4,873 RA cases and 17,642 normal individuals from the East Asian Population (EAP) were included. The meta-analysis resulted in an additional 4 significant SNPs (Table 3; Figure 2) including rs4285314 (miR-3135b, FDR = 1.10 × 10^−13^), rs28477407 (*miR-4308*, FDR = 3.44 × 10^−5^), rs5997893 (*miR-3928*, FDR = 5.9 × 10^−3^) and rs45596840 (*miR-4482*, FDR = 6.6 × 10^−3^). Finally, we also evaluated whether rs1414273 (*miR-548ac*) and rs2620381 (*miR-627*) are significant RA susceptibility variants independent of HLA variants. We found that the association at rs1414273 (OR = 1.18, *p* = 1.03 × 10^−3^) and rs2620381 (OR = 1.31, *p* = 2.55 × 10^−3^) remained significant following adjustment for HLA alleles.

**TABLE 1:** Summary of risk alleles, 1,000 Genome frequencies and SNPs associated with rheumatoid arthritis.

CHR	SNP	A1/A2	RA (no.)	Normal (no.)	OR	P	EAS	EUR	AFR
6p21.32	rs9268839	A/G	42.4% (1,364)	56.0% (1768)	0.58 (0.53–0.64)	3.95 × 10^−27^ ^a^	0.354	0.451	0.236
6p21.32	rs9275376	T/G	25.0% (804)	15.7% (496)	1.79 (1.58–2.03)	2.65 × 10^−20^ ^a^	0.192	0.299	0.285
6q23.3	rs7752903	G/T	6.4% (205)	4.3% (136)	1.51 (1.21–1.89)	2.33 × 10^−4^ ^a^	0.049	0.019	0.056
6p21.33	rs4947332	T/C	2.2% (72)	1.1% (34)	2.11 (1.4–3.18)	2.78 × 10^−4^ ^a^	0.012	0.029	0.094
1p13.1	rs1414273	C/T	40.5% (1,303)	44.7% (1,412)	0.84 (0.76–0.93)	8.26 × 10^−4^ ^a^	0.585	0.140	0.584
15q15.1	rs2620381	C/A	7.9% (254)	10.1% (318)	0.77 (0.65–0.91)	2.55 × 10^−3^ ^a^	0.086	0.004	0.130
14q32.31	rs75330474	T/C	3.8% (121)	5.0% (159)	0.74 (0.58–0.94)	0.0136	0.065	0.003	0.064
18p11.21	rs370878033	G/A	1.7% (54)	2.6% (81)	0.65 (0.46–0.92)	0.01435	0.032	0.001	0.001
7q22.1	rs3823658	A/G	13.4% (432)	11.5% (363)	1.20 (1.03–1.39)	0.01825	0.138	0.138	0.006
1p32.3	rs74085143	A/G	4.0% (130)	5.3% (166)	0.76 (0.6–0.96)	0.0219	0.048	0.022	0.331
6q23.2	rs7740161	T/A	14.3% (459)	16.3% (515)	0.86 (0.75–0.98)	0.0253	0.102	0.347	0.614
3p21.1	rs4687672	A/G	33.7% (1,082)	31.1% (983)	1.12 (1.01–1.25)	0.02914	0.317	0.264	0.202
22q12.2	rs5997893	A/G	46.9% (1,509)	49.5% (1,565)	0.90 (0.82–1.00)	0.03972	0.498	0.667	0.918
19p13.2	rs2967897	T/C	16.8% (541)	15.0% (473)	1.15 (1.00–1.31)	0.04189	0.849	0.686	0.614
15q25.3	rs76468441	T/C	3.2% (104)	2.4% (76)	1.36 (1.00–1.83)	0.0453	0.042	0.054	0.011

a FDR corrections were not shown in the table while only *p* < 2.55 × 10^−3^ is significant in the table with FDR correction. EAS, EUR, and AFR represent allele frequency of A1 in East Asian, European and African populations based on 1,000 Genome dataset. All the OR and *p*-value are referring to A1 while taking A2 as reference. Bayesian logistic regression model adjusted for covariates including gender, age, and BMI, were applied as the statistical approach.

**TABLE 2:** Association statistics for rheumatoid arthritis susceptibility and genetic variants.

Genotypes	RA	Normal	OR (95% CI)	*p*-value
rs9268839, located at 6p21.32, *HLA-DRB9*				
AA	262 (16.30%)	494 (31.27%)	1 (reference)	1.46 × 10^−27^ ^a^
GA	840 (52.27%)	780 (49.37%)	2.03 (1.69–2.44)	
GG	505 (31.43%)	306 (19.37%)	3.11 (2.52–3.85)	
GG vs. GA + AA	505 (31.43%)	306 (19.37%)	1.91 (1.62–2.25)	4.77 × 10^−15^
GG + GA vs. AA	1,345 (83.70%)	1,086 (68.73%)	2.34 (1.96–2.78)	2.01 × 10^−23^
G allele	1850 (57.56%)	1,392 (44.05%)	1.72 (1.56–1.9)	3.86 × 10^−27^
rs9275376, located at 6p21.32, *HLA-DQB1*				
GG	887 (55.20%)	1,128 (71.39%)	1 (reference)	1.26 × 10^−20^ ^a^
TG	636 (39.58%)	408 (25.82%)	1.98 (1.7–2.32)	
TT	84 (5.23%)	44 (2.78%)	2.43 (1.65–3.62)	
TT vs TG + GG	84 (5.23%)	44 (2.78%)	1.93 (1.31–2.86)	5.58 × 10^−4^
TT + TG vs GG	720 (44.80%)	452 (28.61%)	2.03 (1.74–2.35)	2.05 × 10^−21^
T allele	804 (25.02%)	496 (15.70%)	1.79 (1.58–2.03)	2.01 × 10^−20^
rs7752903, located at 6q23.3, *TNFAIP3*				
TT	1,408 (87.62%)	1,445 (91.46%)	1 (reference)	4.19 × 10^−4^ ^a^
GT	193 (12.01%)	134 (8.48%)	1.48 (1.16–1.88)	
GG	6 (0.37%)	1 (0.06%)	6.16 (0.75–283.06)	
GG vs GT + TT	6 (0.37%)	1 (0.06%)	5.92 (0.72–272.02)	0.1246
GG + GT vs TT	199 (12.38%)	135 (8.54%)	1.51 (1.19–1.92)	4.12 × 10^−4^
G allele	205 (6.38%)	136 (4.30%)	1.51 (1.21–1.91)	2.33 × 10^−4^
rs4947332, located at 6p21.33, Tag-SNPs of HLA-DRB1*04:07				
CC	1,537 (95.64%)	1,546 (97.85%)	1 (reference)	5.51 × 10^−4^ ^a^
TC	68 (4.23%)	34 (2.15%)	2.01 (1.31–3.15)	
TT	2 (0.12%)	0 (0.00%)	—	
TT vs TC + CC	2 (0.12%)	0 (0.00%)	—	0.4998
TT + TC vs. CC	70 (4.36%)	34 (2.15%)	2.07 (1.35–3.24)	4.53 × 10^−4^
T allele	72 (2.24%)	34 (1.08%)	2.11 (1.38–3.28)	2.67 × 10^−4^
rs1414273, located at 1p13.1 within *miR-548ac*				
CC	284 (17.67%)	307 (19.43%)	1 (reference)	4.84 × 10^−4^ ^a^
TC	735 (45.74%)	798 (50.51%)	1.00 (0.82–1.21)	
TT	588 (36.59%)	475 (30.06%)	1.34 (1.09–1.65)	
TT vs TC + CC	588 (36.59%)	475 (30.06%)	1.34 (1.15–1.56)	9.35 × 10^−5^
TT + TC vs CC	1,323 (82.33%)	1,273 (80.57%)	1.12 (0.94–1.35)	0.2023
T allele	1911 (59.46%)	1748 (55.32%)	1.18 (1.07–1.31)	9.04 × 10^−4^
rs2620381, located at 15q15.1 within *miR-627*				
CC	9 (0.56%)	20 (1.27%)	1 (reference)	7.27 × 10^−3^ ^a^
AC	236 (14.69%)	278 (17.59%)	1.89 (0.8–4.79)	
AA	1,362 (84.75%)	1,282 (81.14%)	2.36 (1.02–5.91)	
AA vs. AC + CC	1,362 (84.75%)	1,282 (81.14%)	1.29 (1.07–1.56)	7.21 × 10^−3^
AA + AC vs. CC	1,598 (99.44%)	1,560 (98.73%)	2.28 (0.99–5.7)	0.04035
A allele	2,960 (92.10%)	2,842 (89.94%)	1.3 (1.09–1.56)	0.00285

a indicating *p*-values from genotype-based chi-square or Fisher exact test (when N<=5) models. All the OR and *p*-value are calculated compared with homogeneous protective allele (reference row as the table). Chi-square or Fisher exact test was applied as the statistical approach so that ORs are comparative between different models.

**TABLE 3:** Meta-analysis between our study and previous East-Asian population-based RA GWAS study.

CHR	SNP	BP	A1	OR (meta)	P (meta)	FDR
6	rs9268839 (*HLA-DRB9*)	32428772	A	0.52	4.15 × 10^−49^	2.08 × 10^−47^
6	rs9275376 (*HLA*-*DQB1*)	32668633	T	1.72	1.11 × 10^−47^	1.04 × 10^−45^
6	rs7752903 (*TNFAIP3*)	138227364	T	0.73	1.94 × 10^−22^	1.21 × 10^−20^
6	rs4285314 (*miR*-*3135b*)	32717702	A	1.33	2.35 × 10^−15^	1.10 × 10^−13^
14	rs28477407 (*miR*-*4308*)	55344901	T	0.89	9.19 × 10^−07^	3.44 × 10^−5^
22	rs5997893 (*miR*-*3928*)	31556103	A	0.93	1.92 × 10^−04^	5.97 × 10^−3^
10	rs45596840 (*miR*-*4482*)	106028154	A	0.89	2.49 × 10^−04^	6.65 × 10^−3^
6	rs4947332 (*HLA*-*DRB1*)	31834197	T	1.41	9.50 × 10^−04^	2.22 × 10^−2^

### Epistasis Analysis to Identify miRNA Interactions

miRNAs play multiple regulatory roles in both the modification of target gene expression and larger regulatory networks. To identify epistatic effects between miRNAs and their role in the susceptibility of RA, we applied an epistasis analysis to reveal the interaction between the above identified miRNA SNPs. We found 19 SNP-SNP epistatic interactions with *p* < 7.3 × 10^−4^, indicating significant interactions (Supplementary Table S3). We found 10 SNP-SNP pairs that showed a significantly strengthened interaction (OR>1) while nine SNP-SNP pairs showed impaired interaction (OR<1). In addition, enhanced risk epistasis was also identified across HLA alleles (rs4947332 in *HLA-DRB1 and* rs9275376 in *HLA-DQB1*) between HLA and miRNAs (*HLA-DRB1* and *MIR3928*), and between *TNFAIP3* and *MIR5695* (Table 4). A significant positive interaction between rs4947332 (*HLA-DRB1*) and rs5997893 (*MIR3928*) with a significantly inflated OR = 2.83 (95%CI: 1.75–4.58, *p* = 1.36 × 10^−5^, Table 4) for double risk allele carriers, indicating the importance of HLA and non-HLA genetic variation interaction in RA susceptibility.

**TABLE 4:** Epistasis analysis to identify SNP-SNP interaction in rheumatoid arthritis susceptibility.

Epistasis	SNP Genotypes		RA	Control	OR	P
rs4947332 & rs9275376	rs4947332	rs9275376				
	CC	GG	885	1,119	1 (reference)	
	CT + TT	GG	2	9	0.38 (0.1–1.38)	0.164
	CC	GT + TT	652	427	1.93 (1.66–2.24)	6.59 × 10^−18^
	CT + TT	GT + TT	68	25	3.35 (2.12–5.31)	6.70 × 10^−8^
rs4947332 & rs5997893	rs4947332	rs5997893				
	CC	GG	330	392	1 (reference)	
	CT + TT	GG	9	9	1.19 (0.49–2.89)	0.821
	CC	GT + TT	1,207	1,154	1.24 (1.05–1.47)	0.012
	CT + TT	GT + TT	61	25	2.83 (1.75–4.58)	1.36 × 10^−5^
rs7752903 & rs2967897	rs7752903	rs2967897				
	TT	CC	953	1,050	1 (Reference)	
	TG + GG	CC	148	93	1.75 (1.33–2.29)	5.76 × 10^−5^
	TT	CT + TT	455	395	1.27 (1.08–1.49)	0.00370
	TG + GG	CT + TT	51	42	1.33 (0.88–2.01)	0.20705

Five significant epistasis effects were selected to show the increased or decreased risk effect. rs4947332 located in C2 upstream (*HLA-DRB1*); rs9275376 located in *HLA-DQB1* upstream; rs5997893 located in miR-3928; rs7752903 located downstream of TNFAIP3 and rs2967897 located in miR-5695.

### Cumulative Analysis Revealed Increased Risk Effect on Rheumatoid Arthritis

To estimate the combined effect of the 6 risk alleles (rs1414273T, rs4947332T, rs9268839G, rs9275376T, rs7752903G, and rs2620381A) on RA risk, the individual accumulation of risk alleles was treated as an ordinal variable in a logistic regression analysis adjusted by BMI, age and gender. As anticipated, we found that the RA risk showed a positive correlation with the cumulative number of risk alleles (OR = 1.4, *p* = 2.0 × 10^−16^, Z = 12.54, SE = 0.027, Table 5). In our dataset, the largest subgroup of cases carries five risk alleles (25.6% of RA samples) while the largest subgroup of controls carries only 4 risk alleles (29.4% of control samples). The OR for RA status for carriers with eight risk alleles (2.9% of RA population) was 15.38-fold increased over individuals with only 1 risk allele (10.76% of the normal population). Overall, we identified a novel prediction model with HLA and miRNA SNPs for the baseline risk estimation of rheumatoid arthritis.

**TABLE 5:** Cumulative risk effect of rheumatoid arthritis increased as more risk alleles were carried by patients.

No. of risk alleles^a^	RA	Normal	OR (95% CI)	P
1	4	17	1.00 (Reference)	—
2	65	133	1.77 (0.63–4.99)	0.348209
3	206	340	2.19 (0.80–5.97)	0.129698
4	388	466	3.00 (1.10–8.15)	0.031822
5	411	359	4.12 (1.51–11.21)	0.002819
6	344	185	6.68 (2.44–18.27)	6.30 × 10^−05^
7	138	67	7.36 (2.62–20.66)	4.26 × 10^−05^
8	46	10	15.38 (4.69–50.49)	1.00 × 10^−06^

a individuals carrying 0 or more than 9 risk alleles were not found in our dataset. We only found 8 individuals carrying 9 risk alleles (5 RA and 3 normal), which is not stable for the estimation and therefore ignored in this table. rs1414273 (T), rs4947332 (T), rs9268839 (G), rs9275376 (T), rs7752903 (G) and rs2620381 (A) were included in the analysis. Risk alleles are shown in the parentheses following SNPs.

### Regulatory Network Constructed by Significant miRNAs and RA GWAS Associated Genes

**FIGURE 3:**
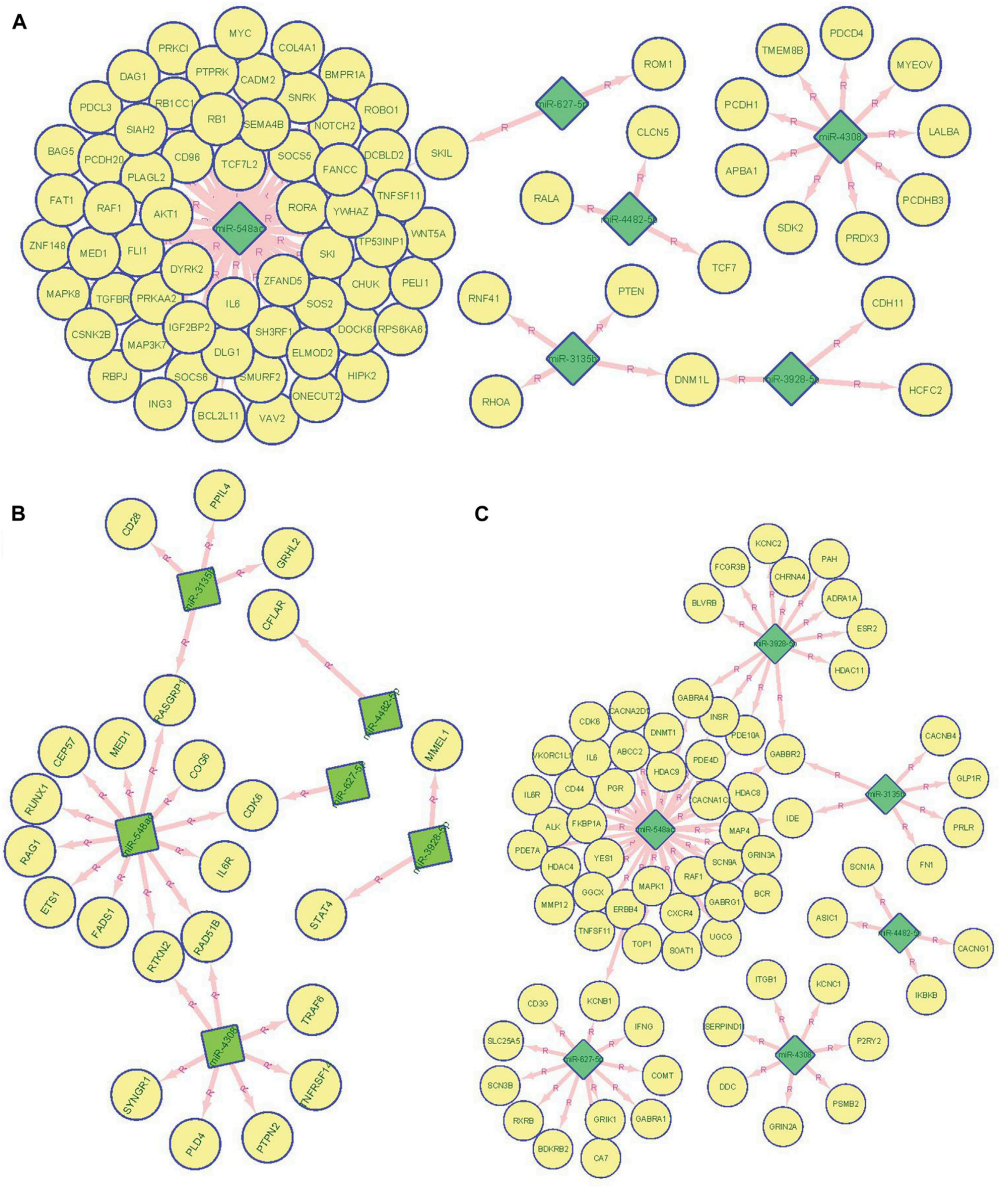
miRNA target analysis and target distribution in GWAS significant genes, immune genes, and FDA drug target genes. **(A)**. Network connection between the top 6 miRNA predicted regulatory targets and previous GWAS identified 123 RA associated genes. **(B)**. Network connection between the top 6 miRNA predicted regulatory targets and 4,723 InnateDB collected immune genes. **(C)** Network connection between the top 6 miRNA predicted regulatory targets and 672 FDA approved drug target genes. miRNA was labeled as diamond filled with green color and gene were as a circle filled with yellow color.

To construct regulatory networks between miRNAs and genes, we predicted target genes for the significant miRNAs identified in our study. First, we predicted candidate genes of our significant miRNAs and then mapping these targets to overall immune related genes collected by InnateDB. We found target genes of RA-associated miRNAs were significantly enriched in the immune related gene category (*p* < 2.2 × 10^−16^, empirical *p* = 2.0 × 10^−5,^ and FC = 1.43, Figure 3A; Supplementary Figure S1A). In addition, we found *miR*-*548ac* has 12 target genes overlapping with previous GWAS (Okada et al., 2014)-identified RA-associated genes including *MED1*, *IL6R*, *CEP57*, *CDK6*, *RAD51B*, *RUNX1*, *RTKN2*, *RAG1*, *FADS1*, *RASGRP1*, *ETS1,* and *COG6*. *miR*-*4308* showed seven candidate genes including *RTKN2*, *TRAF6*, *RAD51B*, *PTPN2*, *PLD4*, *TNFRSF14,* and *SYNGR1*. *miR*-*3135b* showed 4 target genes including *GRHL2*, *CD28*, *PPIL4,* and *RASGRP1*. Although a hypergeometric test showed that miRNA targets (Supplementary Table S4) significantly enriched in GWAS-identified RA candidate genes (*p* = 7.66 × 10^−3^, FC = 1.59, Figure 3B), permutation based analysis showed a non-significant enrichment (empirical *p* = 0.23) indicating an unstable enrichment (Supplementary Figure S1B). Finally, we also observed that miRNA targets were also enriched in FDA-approved drug targets (*p* < 2.2 × 10^−16^, empirical *p* = 0.014, FC = 1.82) indicating miRNA as key regulatory network hubs and that they might be promising drug targets for autoimmune diseases (Figure 3C; Supplementary Figure S1C).

## Discussion

In this study, 225 East-Asian common SNPs located in human microRNA seed regions were genotyped and association and epistasis analyses were conducted to investigate the association between miRNA and seropositive RA in the Han Chinese population (Shanghai, China). To evaluate the potential association of these East-Asian-specific common miRNA SNPs with RA susceptibility, a case/control study was conducted involving 1,607 seropositive RA patients compared to 1,580 matched controls. The study identified 6 novel RA-associated miRNA SNPs (rs1414273 in *miR-548ac*, rs2620381 in *miR-627*, rs4285314 in *miR-3135b*, rs28477407 in *miR-4308*; rs5997893 in *miR-3928* and rs45596840 in *miR-4482*) and revealed the interaction between HLA alleles and miRNA SNPs, thereby advancing understanding of RA pathogenesis.

We found that rs45596840 and rs2620381 located in seed regions of *miR-4482* and *miR-627*, respectively, are significantly associated with RA. rs9268839-G (*HLA-DRB9*) was shown as a risk allele with OR = 2.47 in Caucasian populations and we found that the OR is 1.72, indicating a consistent risk effect that needs to be validated in African populations. According to the miRNA-mRNA binding imputation, rs45596840 (*miR*-*4482*) and rs2620381 (*miR*-*627*) will affect more than 5,894 (2,132 gain and 3,517 loss, see *URL*) and 4,845 (2,593 gain and 2,252 loss, see *URL*) mRNA-miRNA bindings respectively. These miRNA target changes might alter the immune response significantly. In addition, the common regulation network between *miR-4707* and *miR-627* with *CD244*, *CAMTA1* might be of interest. rs1414273 is located within the *miR-548ac* stem-loop sequencing in the first intron of the *CD58* gene, which has shown a strong linkage disequilibrium with the Multiple Sclerosis (MS)-associated haplotype (Hecker et al., 2015). Hecker et al. found that SNP rs1414273 might alter Drosha cleavage activity to provoke partial uncoupling of *CD58* gene expression and microRNA-548ac production from the shared primary transcript in immune cells and regulate the inflammatory processes and the balance of protein folding and degradation (Hecker et al., 2019). However, *CD58*-*CD2* interactions recruiting lymphocytes to inflammatory sites play a crucial role in autoimmune disease (Hoffmann et al., 1996; Sable et al., 2016). Finally, in our previous research, we found that DNA methylation of CD4^+^ cells in rheumatoid arthritis showed abnormal DNA methylation in HLA regions (Guo et al., 2017). The current study found that interactions between HLA-miRNA contributed to RA susceptibility. Overall, the interaction between regulation/epigenetics (Guo et al., 2017) and genetic variation in RA is a promising field for future research.

There are limitations to this research. Due to the scope of the study, only a limited number of ancestry-informative SNPs were used to control for confounding by population stratification. To validate the RA phenotyping, we used 4 East-Asian GWAS RA-associated variants as positive controls for RA samples. All the enrolled individuals were from a three-generation Han Chinese family without genetic admixture with other non-Han ethnic individuals to avoid population stratification. We evaluated the power with Harvard Power Estimation Tool to handle multiple test correction issues with the following parameters: A = 0.05, *p* = 0.01, OR (Aa) = 1.5, OR (AA) = 3, *N* = 1,600, RCC = 1, *p* = 2.2 × 10^−4^. We found that the power for dominant, recessive, and additive models are 0.59, 0.55, and 0.66 respectively. To receive a power of 0.8, the sample size was required to be larger than 2,100, 2,200, and 1,900. Since we undertook an extra meta-analysis where 4,873 RA cases and 17,642 normal are included, we believe this power is enough to make a corresponding conclusion. This study did not provide functional validation of these miRNAs, which is important to show biological validation of the miRNA findings and understand the mechanisms by which these miRNAs are involved in RA susceptibility. Subsequent studies should focus on the functional exploration of these miRNAs. Overall, we identified 6 miRNA SNPs that are significantly associated with the Han Chinese RA population. Two of these SNPs are located in the seed region of their miRNAs and are expected to cause target gain/loss, while another three SNPs are located in the loop/pre-miRNA regions which may influence the stability of corresponding miRNAs.

## Conclusion

Genome-wide association studies have identified several hundred genetic risk factors for rheumatoid arthritis. However, the reported genetic variants only explain less than 40% heritability of rheumatoid arthritis. Hence, most of the heritability for rheumatoid arthritis liability remains missing. Furthermore, heterogeneity in rheumatoid arthritis susceptibility across populations is poorly understood. Interrogation of the genetic architecture of rheumatoid arthritis within non-European-derived populations may provide additional insight into the molecular etiology of the disease, revealing additional pathways that may contribute to the pathogenesis. In this study, we investigated the association between all known common SNPs within East Asian populations located in miRNAs and subsequently tested for the association of these SNPs with rheumatoid arthritis within a well-characterized sample set of Han Chinese origin (1,625 RA and 1,598 controls). We found two significant miRNA SNPs (rs1414273 in *miR*-*548ac* and rs2620381 in *miR-627*) significantly associated with RA in our dataset. A meta-analysis with previous GWAS studies, including 4,873 RA cases and 17,642 controls, discovered another 4 novel miRNA RA-associated SNPs (rs4285314 in *miR-3135b*, rs28477407 in *miR-4308*, rs5997893 in *miR*-*3928,* and rs45596840 in *miR*-*4482*). In addition, we identified numerous HLA-HLA and HLA-miRNA epistatic effects, indicating the importance of interactions between genetic variants in HLA and miRNA systems in susceptibility to RA. We found that individuals who carried 8 risk alleles were estimated to be at 15.38-fold increased risk of RA. Finally, we found that the targets of the significant miRNAs showed enrichment in immune related genes and FDA approved drug target genes, indicating miRNAs might be interesting targets to accelerate understanding of the pathogenesis and drug development for rheumatoid arthritis. Our study suggested that the interaction between HLA and functional SNPs in human regulatory elements may be a fruitful avenue of investigation in further understanding susceptibility to RA.

## Data Availability

The original contributions presented in the study are included in the article/Supplementary Material, further inquiries can be directed to the corresponding authors.
