# Supplementary figures and images for: MicroRNA Variants and HLA-miRNA Interactions are Novel Rheumatoid Arthritis Susceptibility Factors
Source: Front Genet. 2021 Oct 29;12:747274. doi: 10.3389/fgene.2021.747274 (PMC8585984; doi:10.3389/fgene.2021.747274)

A

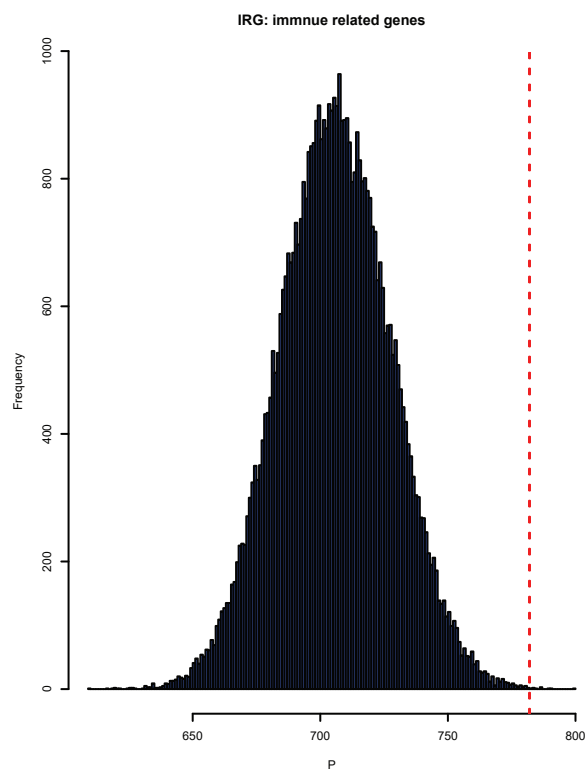

B

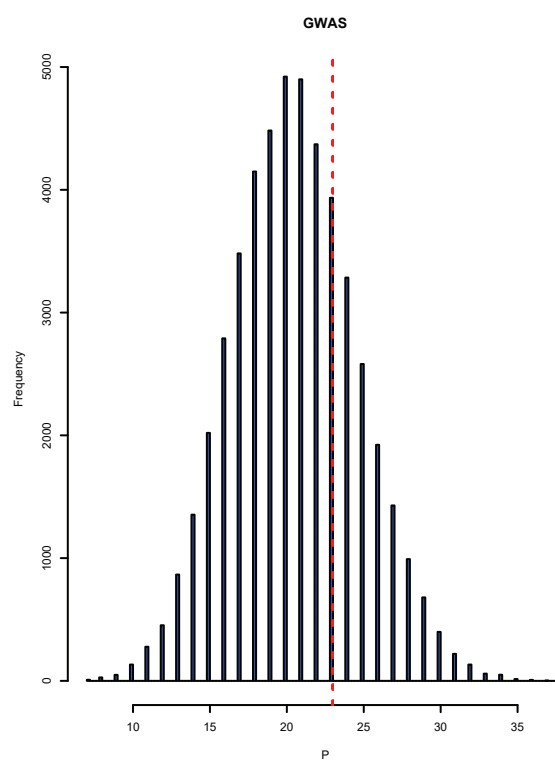

C

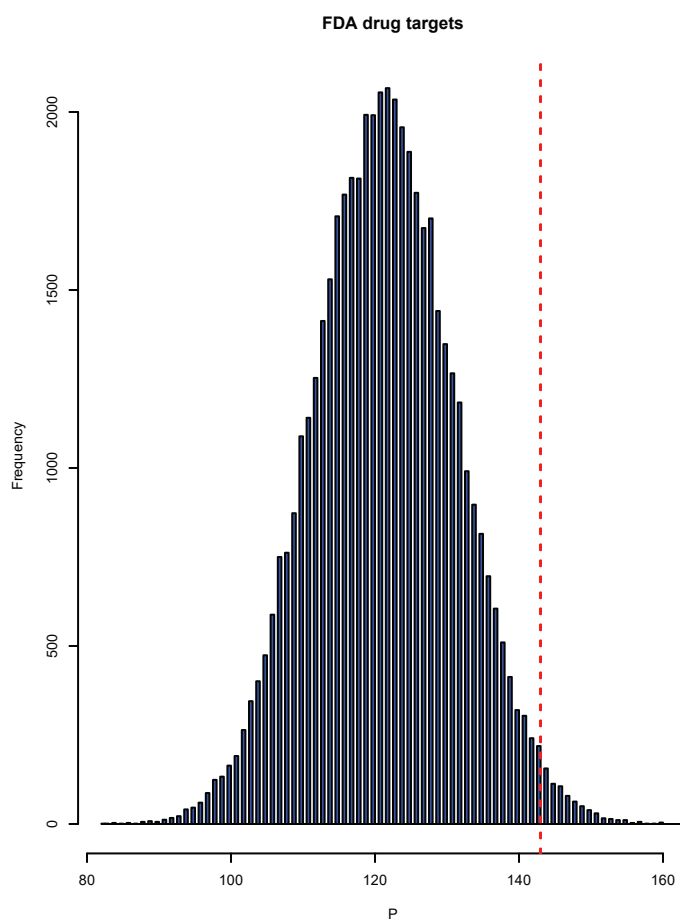

Supplement: Supplementary file 5 [file Image1.pdf]
